# Radiolabeling of Nucleic Acid Aptamers for Highly Sensitive Disease-Specific Molecular Imaging

**DOI:** 10.3390/ph11040106

**Published:** 2018-10-15

**Authors:** Leila Hassanzadeh, Suxiang Chen, Rakesh N. Veedu

**Affiliations:** 1 Department of Nuclear Medicine, School of Medicine, Rajaie Cardiovascular, Medical and Research Center & Department of Medicinal Chemistry, School of Pharmacy-International Campus, Iran University of Medical Sciences, Tehran 1449614535, Iran; hassanzadeh.l@iums.ac.ir; 2 Centre for Comparative Genomics, Murdoch University, Perth 6150, Australia; S.Chen@murdoch.edu.au; 3 Perron Institute for Neurological and Translational Science, Perth 6009, Australia

**Keywords:** aptamers, aptamer-targeted imaging, molecular imaging, aptamer-radiolabeling

## Abstract

Aptamers are short single-stranded DNA or RNA oligonucleotide ligand molecules with a unique three-dimensional shape, capable of binding to a defined molecular target with high affinity and specificity. Since their discovery, aptamers have been developed for various applications, including molecular imaging, particularly nuclear imaging that holds the highest potential for the clinical translation of aptamer-based molecular imaging probes. Their easy laboratory production without any batch-to-batch variations, their high stability, their small size with no immunogenicity and toxicity, and their flexibility to incorporate various functionalities without compromising the target binding affinity and specificity make aptamers an attractive class of targeted-imaging agents. Aptamer technology has been utilized in nuclear medicine imaging techniques, such as single photon emission computed tomography (SPECT) and positron emission tomography (PET), as highly sensitive and accurate biomedical imaging modalities towards clinical diagnostic applications. However, for aptamer-targeted PET and SPECT imaging, conjugation of appropriate radionuclides to aptamers is crucial. This review summarizes various strategies to link the radionuclides to chemically modified aptamers to accomplish aptamer-targeted PET and SPECT imaging.

## 1. Introduction

Molecular imaging technologies employ labeled molecules to explore biological targets in living subjects for disease detection and monitoring treatment progress in real time. The nuclear imaging technique is a powerful approach to achieve highly sensitive imaging. The two most current approaches, that is, positron emission tomography and single photon emission computed tomography, use radionuclides to label specific therapeutic and/or diagnostic (theranostic) molecules for diagnosis and prognosis of many diseases [1,2]. Clinical molecular imaging using these techniques facilitates characterization of biological processes in vivo on a molecular and cellular level [3]. In modern nuclear medicine, approximately 95% of radiopharmaceuticals are used for diagnostic purposes [4]. In addition to small chemical structures, radiopharmaceuticals may consist of bigger biomolecules, such as antibodies, antibody fragments, proteins, peptides, and nucleic acids that are usually more specific [5].

**Figure 1:**
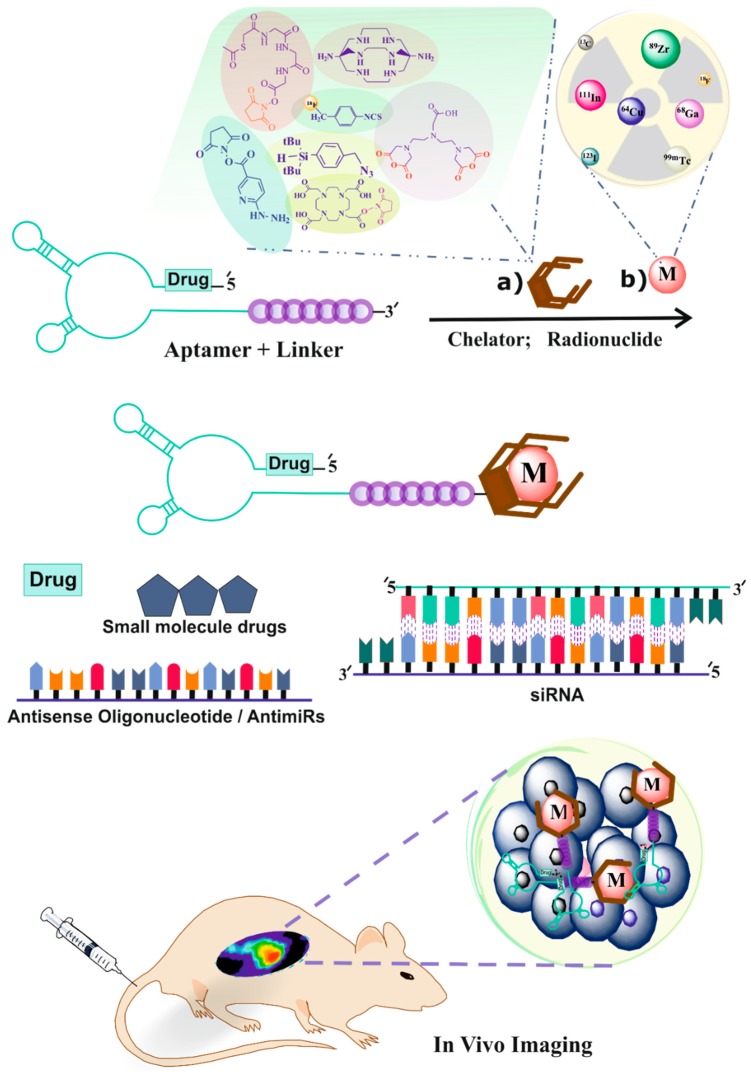
Schematic presentation of aptamer-based therapeutic and/or diagnostic (theranostic) platform technology for targeted therapy and molecular imaging applications.

Aptamers are oligomers composed of deoxyribonucleotides (DNA aptamers) or ribonucleotides (RNA aptamers), which are promising targeting biomolecules in nuclear medicine. Various three-dimensional structures of aptamers via intramolecular interactions, such as hydrogen bonds, hydrophobic effects, Vander Waals forces, and so on, enable them to bind the target binding domain (aptatope) [6]. Aptamers are able to bind different types of targets, such as small ions like Zn^2+^, large targets like proteins, whole cells, bacteria, or viruses [7,8,9,10]. These structures can easily differentiate between closely similar molecules, such as the presence or absence of a hydroxyl or methyl group, a urea against a guanidine group, and d or l amino acids that make them highly specific compounds [11,12,13,14]. Their high specificity, easy solid phase chemical synthesis, and small size for high tissue penetration make aptamers excellent alternate molecules for use in the medical, pharmaceutical, and biotechnological fields [15]. In this review, we focus on various methods of radiolabeling that have been performed using aptamers and other potential approaches to producing diagnostic tracers in SPECT and PET imaging techniques (Figure 1).

## 2. Improving the Stability of Aptamers

**Figure 2:**
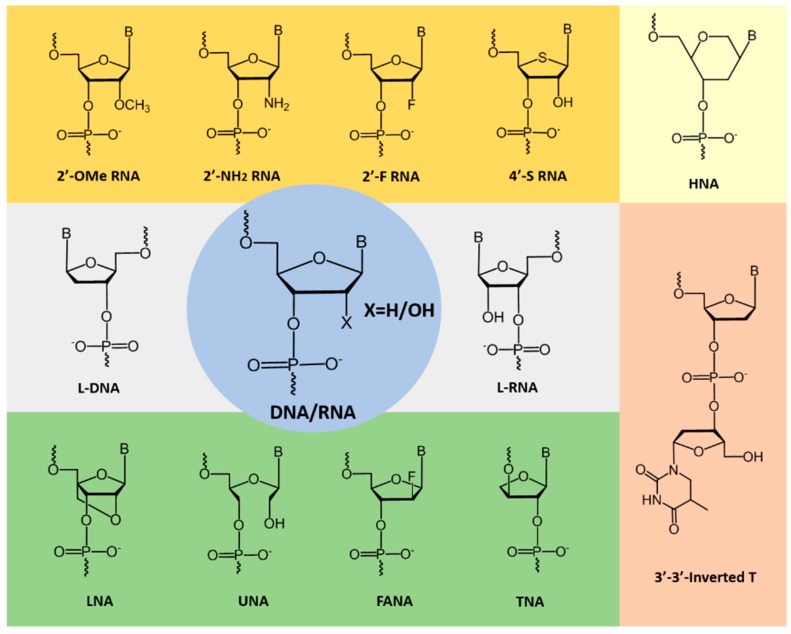
Examples of chemically-modified nucleotides used to improve aptamer stability and kinetics.

Nucleic acid aptamers composed of natural nucleotide monomers show very low resistance to nucleases and are rapidly degraded in vivo. To overcome this significant limitation, chemically-modified nucleotide analogues (Figure 2) are generally systematically incorporated to a developed DNA or RNA aptamer without compromising the target binding affinity and specificity [16]. Some of the prominent modified nucleotides which have been explored in aptamer fabrication are shown in Figure 2. Recently, Lipi et al. provided an in-depth review on developing chemically-modified aptamers [17]. Often termed as ‘chemical antibodies’, aptamers are ideal alternative molecules to antibodies for targeted molecular imaging applications, and several researchers utilized the potential of aptamers in nuclear medicine [18]. Different factors influence the in-vivo half-life of the radiolabeled aptamers and alter the biodistribution patterns and clearance pathways, which greatly influence imaging efficiency. Fast clearance of imaging probes might be an advantage because of high target to background ratio. Incorporation of chemically-modified nucleotides certainly improves the properties of aptamers as targeted imaging probes; however, improved nuclease resistance and stability could also enhance the retention time of aptamers and target binding in the body [19].

## 3. SPECT and PET Imaging Techniques

Based on the type of radionuclides used, two types of imaging techniques are employed in nuclear medicine, (1) SPECT (single-photon emission computed tomography), which employs γ-emitting radionuclides, including ^99m^Tc, ^123^I, ^67^Ga, and ^111^In, and (2) PET (positron emission tomography), which uses β^+^-emitting radionuclides, including ^18^F, ^11^C, ^13^N, ^15^O, ^68^Ga, ^82^Rb, ^64^Cu, and ^89^Zr [4]. Table 1 shows the properties of prominent radionuclides, all of which are diagnostic radionuclides and emit a positron or gamma photon. Each radionuclide has a specific physical half-life, decay mode, chemical properties, and a production method. The physical half-life and decay mode is characteristic for a given radionuclide and not aligned with any physicochemical condition and cannot be changed with any other method, such as a physicochemical modification. Therefore, it is important to consider the characteristics of radionuclides to target the biological process or disease, which is to be visualized, characterized, or measured [20].

## 4. Radiolabeling of Ligands for SPECT Imaging

### 4.1. ^99m^Tc-Radiolabeling

Currently, in most diagnostic nuclear medicine procedures ^99m^Tc is used because of its desirable physical characteristics (half-life: 6.02 h; energy: 140.51 keV and pure gamma emission). Additionally, technetium-99m is readily available in a sterile, pyrogen-free and carrier-free state from ^99^Mo/^99m^Tc generators. On the other hand, radiolabeling of aptamers in most situations is a challenging process that needs further chemical synthesis in order to conjugate aptamers to chelating agents, such as Mercaptoacetyltriglycine (MAG_3_), Diethylenetriaminepentaacetic acid (DTPA), Hydrazinonicotinamide (HYNIC), and Tetraazacyclododecane tetraacetic acid (DOTA), which have the capability for radioisotopes to be attached to them [21,22]. There are two different methods of ^99m^Tc-labeling for aptamers: (1) Direct method; (2) Indirect method.

#### 4.1.1. Direct ^99m^Tc/^188^Re Radiolabeling of Aptamers

In this method of radiolabeling, ^99m^Tc without any chelating agent attaches to the biomolecule. It seems to be more favorable because of easy and rapid preparation, less cost and manipulation, and reduction of probable degradation. In 2014, for the first time, the direct ^99m^Tc-labeling of aptamers was reported by Correa C.R. et al. [23]. Two aptamers, with and without primary amine group, were selected and radiolabeled with ^99m^TcO_4_^−^ in the presence of stannous chloride as a reducing agent. The reduction of ^99m^Tc from 7+ oxidation state to a lower oxidation state is required to stimulate a chemical reaction of the ^99m^Tc to many compounds [4]. The aforementioned aptamers were directly labeled with ^99m^Tc by addition of an aliquot of Na^99m^TcO_4_ in the sealed vial, containing Ethylenediamine-*N*,*N*′-diacetic acid (EDDA) and tricine. Both aptamers’ radiolabeling showed high radiochemical efficiencies and considerable in vitro stability [23]. The next direct radiolabeling of aptamers was performed on several unmodified aptamers with an amine-ended linker by Cao et al. in 2015, and on (1→3)-β-D-glucan aptamers by de Sousa Lacerda et al. in 2017 [24,25]. Radiochemical purities that were reported by TLC (thin layer chromatography) indicated high radiolabeling efficiency. Direct radiolabeling of aptamers with Rhenium (^188^Re) was reported to radiolabel a DNA aptamer to target EGFRvIII for glioblastoma diagnoses by Xidong Wu et al. in 2014. In this method, an aptamer was dissolved in acetic acid (pH 5.0) and radiolabeling was performed in the presence of SnCl_2_ and ascorbic acid, followed by the addition of ^188^Re eluent and warming at 37 °C for 1.5 h [26].

#### 4.1.2. Indirect ^99m^Tc-Labeling of Aptamers

The labeling of oligomers is performed using a similar strategy to that employed for other biologicals, such as antibodies and peptides. Since ^99m^Tc is an isotope of a metal, the chelator molecule needs to be attached via a linker to avoid steric hindrances [27]. The ^99m^Tc species in a lower oxidation state react chemically with a different type of chelating agents. The chelating agent usually donates lone pairs of electrons to reduced ^99m^Tc to generate coordinate covalent bonds [4]. The application of bifunctional chelators such as MAG_3_, DTPA, and DOTA for radiolabeling oligonucleotides with some radionuclides is described below [27].

**Figure 3:**
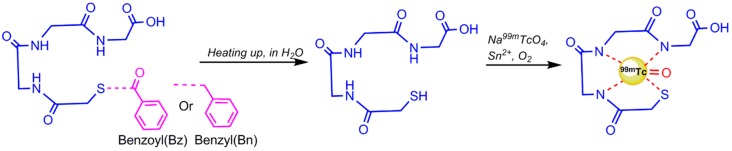
^99m^Tc radiolabeling of S-benzoyl Mercaptoacetyltriglycine (MAG_3_) chelator.

*Mercaptuacetyltriglycine (MAG_3_) derivatives:* One of the attractive ^99m^Tc-chelators for labeling biomolecules is MAG_3_ that forms stable radioligands in vivo and in vitro without any coligand requirements [28]. Prior to radiolabeling, the tetradentate mercapto–acetyltriglycine is used as its benzyl or benzoyl protected derivative (S-benzoyl or benzyl MAG_3_) to avoid the formation of considerable amounts of disulphide-bond, which is removed by heating at 100 °C for 10 min during ^99m^Tc-labeling (Figure 3) [29,30]. Rapid deprotection requires higher temperature and alkaline pH [31].

**Figure 4:**
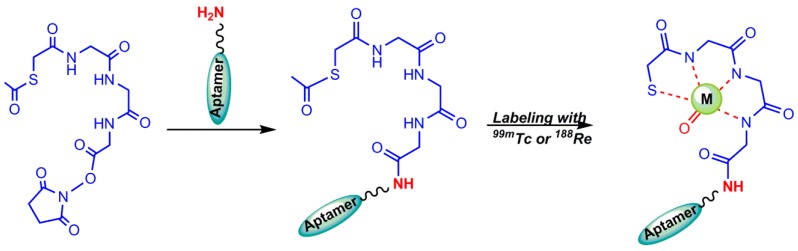
Conjugation of the NHS–MAG_3_ chelator to an amine-derivatized aptamer, and radiolabeling.

To avoid the tough conditions of alkaline pH and boiled water temperature of benzoyl deprotection, *N*-hydroxysuccinimidyl S-acetylmercaptoacetyltriglycinate (NHS–MAG_3_) was synthesized, in which an acetyl group substituted the benzoyl group. Acetyl as a better leaving group, can be removed at room temperature and neutral pH more easily [32]. The developed MAG_3_ bifunctional chelator (S-acetyl NHS–MAG_3_) was used for ^99m^Tc-radiolabeling of aptamers and amine derivatized oligonucleotides (Figure 4) [27].

However, in line with previous reports, postlabeling purification was required to increase the radiochemical purity to 90% or higher, while the labeling of DNAs was performed with ^99m^Tc at neutral pH and room temperature [31,33,34,35]. But, Wang et al. reported the radiolabeling procedure of a 16-mer oligonucleotide with a primary amine group at 5′-end and NHS–MAG_3_ as a bifunctional chelating agent without any purification requirements after labeling [28]. Using NHS–MAG_3_ as bifunctional chelating agents for ^99m^Tc radiolabeling of different types of oligonucleotides has been reported in various studies [28,36,37,38,39]. The synthesis of NHS–MAG_3_ could be readily conducted according to the procedure described by Winnard et al. [31] or by reacting S–acetylmercaptoacetyltriglycine with *N*–hydroxysuccinimide in the presence of *N*,*N*′–dicyclohexylcarbodiimide (DCC) as a dehydration reagent in anhydrous *N*–methyl-2–pyrrolidinone (NMP). The conjugation procedure of biomolecule to NHS–MAG_3_ and ^99m^Tc-radiolabeling is reported in detail by Wang et al. [28]. A truncated modified RNA aptamer with high affinity for the human matrix metalloprotease 9 (hMMP-9), has been functionalized by conjugating its 5′-end with S–acetylmercaptoacetyltriglycine (MAG_3_) through a hexylamino linker. It was then radiolabeled with ^99m^Tc and successfully employed for the detection of hMMP-9 (the tumor biomarker) in human glioblastoma sections (in vitro) and in mice bearing human melanoma tumors (in vivo) [40,41].

**Figure 5:**
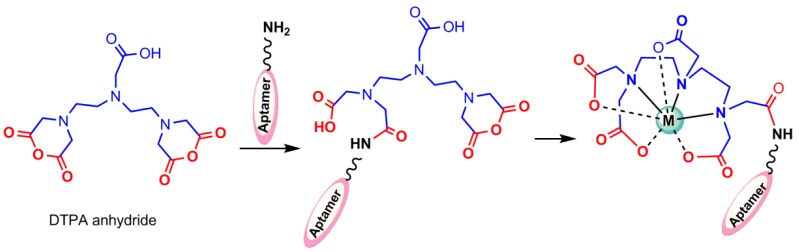
Conjugation of the diethylenetriaminepentaacetic acid (DTPA) anhydride to an amine-derivatized aptamer, and radiolabeling.

*Diethylenetriaminepentaacetic acid (DTPA):* The unshared pair of electrons in the molecules containing NH_3_, −CN, −SH, −COO, −NH_2_, and CO can be donated to a metal ion to form a complex. Diethylenetriaminepentaacetic acid (DTPA) and Ethylenediaminetetraacetic acid (EDTA) are usual examples of such chelating agents, with oxygen in carboxyl groups and nitrogen in the amino groups as donor atoms. DTPA has previously been used for radiolabeling antibodies with ^99m^Tc, but the resulting radiolabeled ligand was considerably unstable [42]. A covalent coupling method of DTPA to proteins using bicyclic anhydride of DTPA has been reported [43], which was prepared by a one-step synthesis according to the method of Eckelman et al. [44]. The product, when protected from moisture, was stable for many months at room temperature, and the cyclic nature of DTPA anhydride produced by the suggested method above was confirmed by the characteristic evaluation [43]. The cyclic anhydride DTPA has been widely used [43] to radiolabel amine derivatized oligonucleotides with ^99m^Tc (Figure 5) [35,45]. An extra amount of anhydride is usually necessary to compensate the hydrolysis of the anhydride that will occur in an aqueous solution in competition with conjugation. DTPA conjugation to DNAs according to the abovementioned method was reported by Hnatowich et al. in 1995 [46].

**Figure 6:**
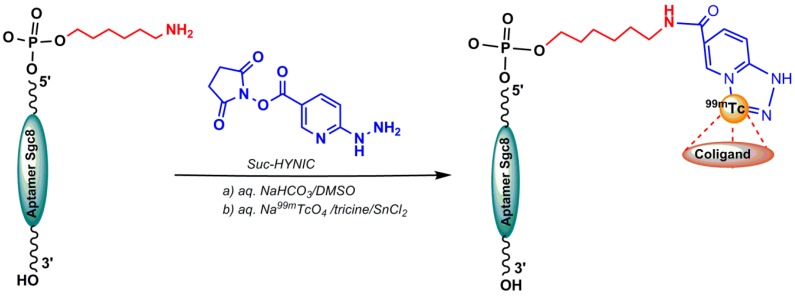
Conjugation of the 6-hydrazinonicotinamide succinimidyl ester (Suc−HYNIC) to an amine-derivatized aptamer, and radiolabeling.

*Hydrazinonicotinamide (HYNIC):* One of the most useful bifuctional chelating agents used in ^99m^Tc-labeling of biomolecules is HYNIC (6-hydrazinopyridine-3-carboxylic acid or hydrazinonicotinamide). The active ester of HYNIC is used to conjugate with the biomolecule—for example, the amino groups of lysine residues in proteins, peptides, or amine-labeled oligonucleotides. HYNIC is able to occupy just two sites of the technetium coordination sphere; coligands such as tricine, EDTA, trisodium triphenylphosphinetrisulfonate (TPPTS) are used to bind other sites and enhance the labeling yield [4]. In 2017, Calzada et al. published a HYNIC-conjugated aptamer radiolabeled with ^99m^Tc. A shortened sequence of the original aptamer Sgc8, which has showed similar binding properties to protein kinase 7 membrane receptor, was modified at the 5′-end with an aminohexyl−moiety (Sgc8−C6−NH_2_) that could be reacted with the bifunctional agent 6-hydrazinonicotinamide succinimidyl ester (Suc−HYNIC) (Figure 6). To synthesize a HYNIC conjugated aptamer, excess amount of Suc−HYNIC (in dry DMSO) was added to an aqueous solution of Sgc8−C_6_−NH_2_. After purification and chemical characterization, ^99m^TcO_4_^−^ was used for radiolabeling the HYNIC chelator by incubating with SnCl_2_ and tricine [47]. Another HYNIC conjugation was reported for the AS1411 aptamer targeting nucleolin protein (dissolved in a borate buffer) using NHS−HYNIC (in dried dimethylformamide). A purified HYNIC aptamer was dissolved in ammonium acetate and radiolabeled by adding ^99m^Tc–pertechnetate solution in the presence of SnCl_2_.2H_2_O as the reducing agent and tricine as a coligand [48].

**Figure 7:**
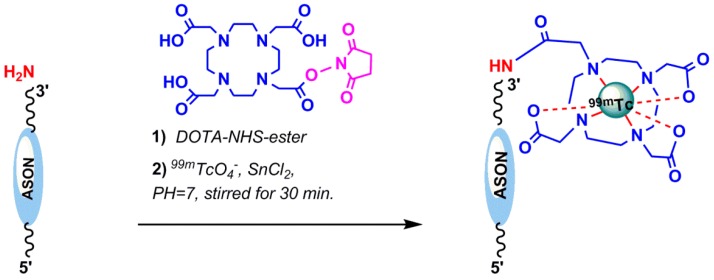
Conjugation of the Tetraazacyclododecane tetraacetic acid (DOTA)–NHS–ester to amine-functionalized oligonucleotide for ^99m^Tc radiolabeling.

*1,4,7,10-Tetraazacyclododecane or 1,4,7,10-tetraacetic acid or Tetraxetan (DOTA):* DOTA, a macrocyclic bifunctional chelating agent, binds to trivalent metals such as Indium (In^3+^), Yttrium (Y^3+^), and other metals of lanthanide series rapidly under mild conditions that enable high radiolabeling efficiencies to be achieved at room temperature in comparison with acyclic ligands, such as DTPA. It can be readily modified into a bifunctional chelating agent by the derivatization of one of the carboxylates [49]. Macrocyclic complexes are often extremely resistant to dissociation (macrocyclic effect) because the structures are rather rigid and a great deal of strain in the ligand may need to be overcome in order to break the first metal–donor bond [50]. In 2012, Ren et al. reported the ^99m^Tc radiolabeling of hTERT (human tumor telomerase reverse transcriptase) antisense oligonucleotide (ASON) through the bifunctional chelator DOTA. The ASON sequence, 5′-TAG AGA CGT GGC TCT TGA-3′, was conjugated with DOTA–NHS via the amine-functionalization of the oligonucleotides at the 3′-end [51]. DOTA–NHS–ester was prepared using the published procedure [52,53]. In brief, DOTA, 1-(3-Dimethylaminoptoply)-3-ethylcarbodimide hydrochloride (EDC–HCl) and *N*–hydroxysulfo–succinimide sodium S–NHS were added in PBS (Phosphate Buffered Saline) (pH 5.5). Then, the semistable amino-reactive intermediate DOTA–NHS–ester was added to ASON and radiolabeled by ^99m^TcO_4_^−^ with SnCl_2_ at room temperature (Figure 7). The reported radiochemical purity was 85% [51]. Calzada et al. successfully linked both ^99m^Tc and ^67^Ga to an aptamer (Sgc8) through the HYNIC and DOTA chelators, which showed desirable biodistribution and pharmacokinetic properties [47]. In 2018, Dr. Hugo Cerecetto et al. reported the synthetic efforts to prepare conjugates between Sgc8-c (a truncated sequence of the original aptamer Sgc8) and different metallic ion chelator moieties, such as HYNIC, DOTA, and NOTA (1,4,7-triazacyclononane–*N*,*N*′,*N*′′–triacetic acid), were covalently attached at the 5′-end of the aptamer, and were found to be stable in an aqueous solution up to 75 °C for 30 days [54].

## 5. Radiolabeling of Ligands for PET Imaging

PET imaging is a sensitive, noninvasive nuclear medicine imaging technique. This technique is able to measure biomedical, pharmacological, and metabolic processes quantitatively in vivo. Consequently, labeling of oligonucleotide with PET radionuclides is a versatile tool to assess the in vivo behavior. Commonly used PET radionuclides are ^15^O, ^13^N, ^11^C, and ^18^F, with 2.07, 9.97, 20.3, and 110 min half-life, respectively, out of which ^18^F has a more favorable half-life [55,56]. Other positron emitting radionuclides are ^64^Cu, ^89^Zr, and, most importantly, ^68^Ga, which is easily available via a Germanium-68/Gallium-68 Generator in nuclear medicine centers.

### 5.1. ^18^F Radiolabeling

#### 5.1.1. ([^18^F]fluoromethyl)phenyl Isothiocyanate

**Figure 8:**
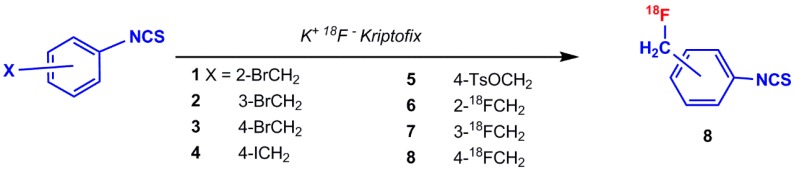
Synthesis of (fluoro–^18^F) ethane isothiocyanatobenzene by various substrates (1–8).

The possibility of incorporating ^18^F into oligonucleotide was reported by Hedberg and Längström in 1997. They developed a one-step synthesis of a labeled compound containing the isothiocyanate functionality ([^18^F]fluoromethyl)phenyl isothiocyanate) and investigated the reaction of ^18^F-labeled oligonucleotide at 5′-position with a hexylamine linker [57]. The mentioned precursors (1–8) in Figure 8 have been prepared by following the procedure described in the literature [55,57,58,59]. Isothiocyanates containing leaving groups, such as bromide, iodide, and tosyl, were used as substrates in nucleophilic substitution reactions with [^18^F]fluoride to produce compound 8 (Figure 8) [57].

**Figure 9:**
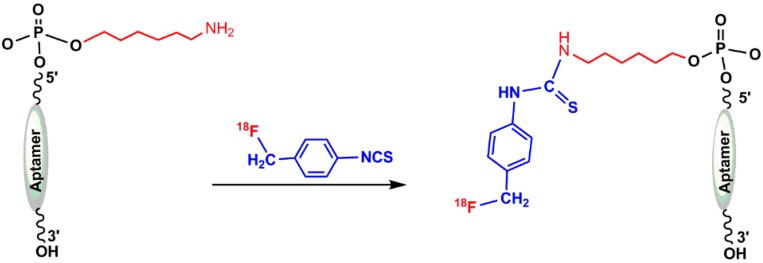
Radiolabeling of 5′-position modified oligonucleotide by ([^18^F]fluoromethyl)phenyl isothiocyanate.

Oligonucleotides can be radiolabeled at 5′-position using ([^18^F]fluoromethyl)phenyl isothiocyanate, as shown in Figure 9. In 2003, de Vries et al. reported the ^18^F-radiolabeling of ASON using different alkylating agents, and *N*–(4-[^18^F]fluorobenzyl)–2-bromoacetamide α–bromo–α’–[^18^F]fluoro–m–xylene were found to be the most promising [56].

#### 5.1.2. Labeling via Click Chemistry Using 1-(Azidomethyl)–4-(Fluoro–^18^F) Benzene

**Figure 10:**
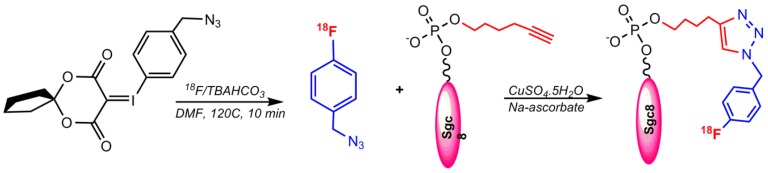
Conjugation of 1-(azidomethyl)–4-(fluoro–^18^F)benzene to a terminal hexynyl modified aptamer.

Another reported method for radiolabeling ^18^F to aptamers is through the click chemistry approach. Radiolabeling of an Sgc8 aptamer targeting PTK7 was performed using an alkynyl functionalized Sgc8 aptamer and ^18^F–benzylazide via click chemistry. The Sgc8 aptamer was modified at the 5′-end with a terminal hexynyl group and the automated radiochemical synthesis of ^18^F–fluorobenzyl azide was achieved using an aromatic fluoride substitution on a spirocyclic hypervalent iodine(III) precursor (Figure 10) [60,61].

The abovementioned click reaction protocol was applied for the PET imaging detection of HER_2_ in a mouse model with ovarian cancer in 2017 by ^18^F radiolabeling of a trivalent HER_2_-targeting aptamer and the alkyne-modified EGFR aptamer (ME07) via click chemistry by Cheng S. et al. in 2018 [62]. The ^18^F–benzyl azide precursor has recently indicated robust and reliable radiolabeling with high radiochemical yield [63,64].

#### 5.1.3. Silicon-Based Chemistry for ^18^F-Radiolabeling

**Figure 11:**
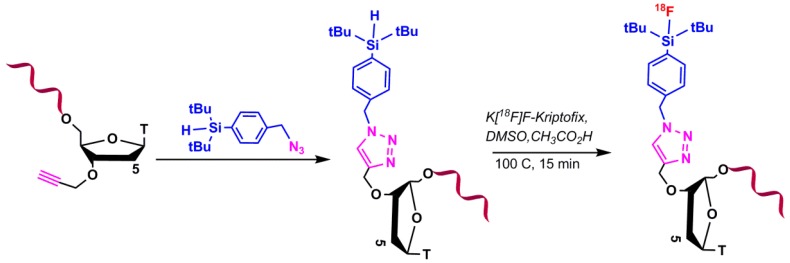
Conjugation of ^18^F by substitution of hydrogen atom on silylated oligonucleotide.

In this method, silylated oligonucleotide was utilized into the ^18^F-fluorination reaction (Figure 11). The silylated oligonucleotides were conjugated to different bifunctional silicon building blocks via click chemistry. Under physiological conditions, the di-tertbutylsilyl group is able to be substituted on the silicon atom to stabilize the SiF bond [65,66].

#### 5.1.4. *N*–Succinimidyl 4-^18^F–Fluorobenzoate (^18^F–SFB)

**Figure 12:**
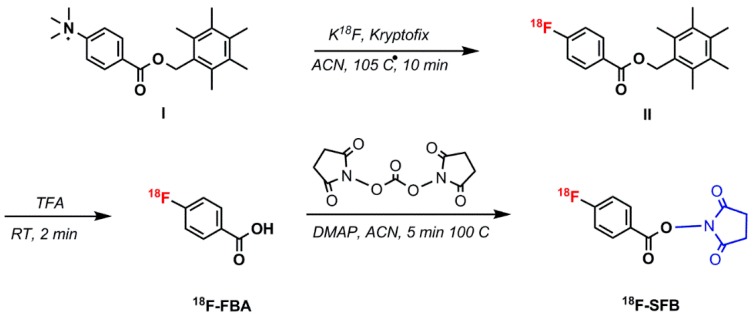
The 3 step synthesis of 2,5-dioxopyrrolidin–1-yl 4-(fluoro–^18^F)benzoate (^18^F–SFB).

**Figure 13:**
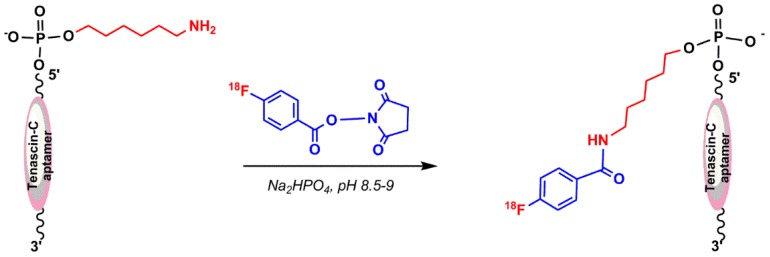
Conjugation of ^18^F–SFB to a Tenascin-C aptamer.

In this method, 2,5-dioxopyrrolidin–1-yl 4-(fluoro–^18^F)benzoate (^18^F–SFB) as a precursor of ^18^F were used to react with a primary amine functionalized aptamer. In 2015, Jacobson et al. reported ^18^F-radiolabeling of a single-stranded DNA aptamer containing 70 nucleotides (Tenascin-C aptamer) using ^18^F–SFB. The aptamer was modified to have a 6-carbon chain and only one primary amine group at the 5′-position for conjugation purposes [67]. Radio-synthesis of ^18^F–SFB was performed in three automated steps using a 2-reaction-vial module. ^18^F–fluoride substitution on *N*,*N*,*N*–trimethyl–4-(2,3,4,5,6-pentamethylbenzyl)oxy)carbonyl)benzenaminium (I) was applied in the first reaction vial. The 4-(fluoro–^18^F)benzoic acid (^18^F–FBA) was obtained by hydrolysis reaction of 2,3,4,5,6-pentamethylbenzyl 4-(fluoro–^18^F)benzoate (II), followed by coupling with bis(2,5-dioxopyrrolidin–1-yl) carbonate and 4-(dimethylamino)pyridine in the second reaction vial to prepare ^18^F–SFB (Figure 12) [68]. The conjugation of ^18^F–SFB to an aptamer was performed in a basic sodium phosphate buffer (Figure 13), which resulted in a low but usable yield [67].

#### 5.1.5. Hybridization-Based ^18^F-Radiolabeling of Aptamers:

**Figure 14:**
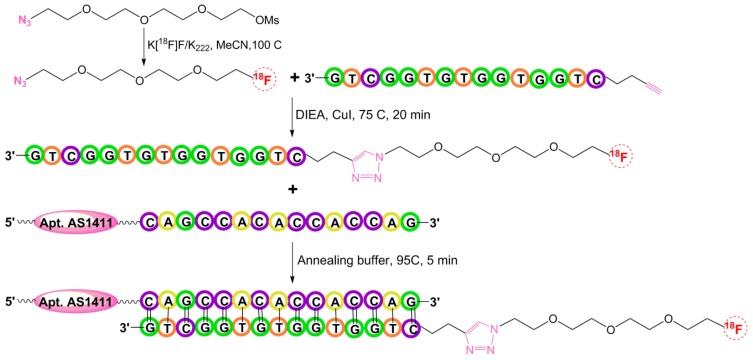
Schematic illustration of Hybridization-based ^18^F-Radiolabeling.

Aptamers or aptamer-containing oligonucleotide sequences can be annealed to complementary sequences by following Watson and Crick base-pairing interactions. Park et al. in 2016 reported the development of the complementary oligonucleotide (cODN) hybridization-based aptamer conjugation approach for aptamer-based molecular imaging. The cODN was prelabeled with ^18^F and hybridized with a complementary sequence containing the AS1411 aptamer sequence in an aqueous condition. Briefly, the precursor PEG–mesylate was added to the ^18^F–KF–K_222_ complex and the mixture was stirred at 100 °C for 10 min. Isolated ^18^F–PEG–Azide (^18^F–FPA) was reacted with a mixture of 5′-alkyne modified oligonucleotide using *N*,*N*–diisopropylethylamine and copper (I) iodide in acetonitrile at 70 °C for 20 min. Then, a fully matched sequence (5′-CAG CCA CAC CAC CAG-3′) containing the nucleolin aptamer AS1411 sequence was hybridized in an annealing buffer by incubating the mixture at 95 °C for 5 min (Figure 14) [69].

### 5.2. ^64^Cu-Radiolabeling

**Figure 15:**
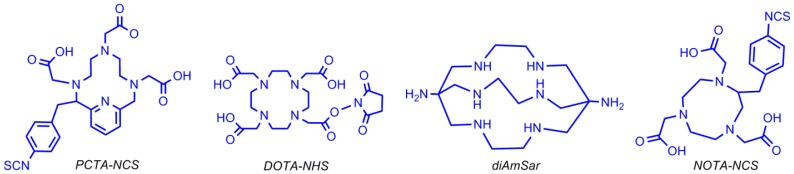
Chelator structures used to conjugate ^64^Cu to biomolecules.

^64^Cu (t_1/2_ = 12.7 h) displays both β-minus decay and positron emission and can be produced with highly specific activity using a medical cyclotron [70,71,72]. The half-life of this radionuclide enables centralized production and purification for research and clinical studies of targeting molecules, such as peptides and aptamers [73,74,75]. The optimal labeling with ^64^Cu can be achieved under physical conditions in which the oligonucleotides are stable. Based on the chemical properties, ^64^Cu is an attractive radionuclide for high-resolution, targeted aptamer-based PET imaging agents [76,77]. In order to radiolabel an aptamer with ^64^Cu, it must be linked to a chelator moiety, as shown in Figure 15 [78].

**Figure 16:**
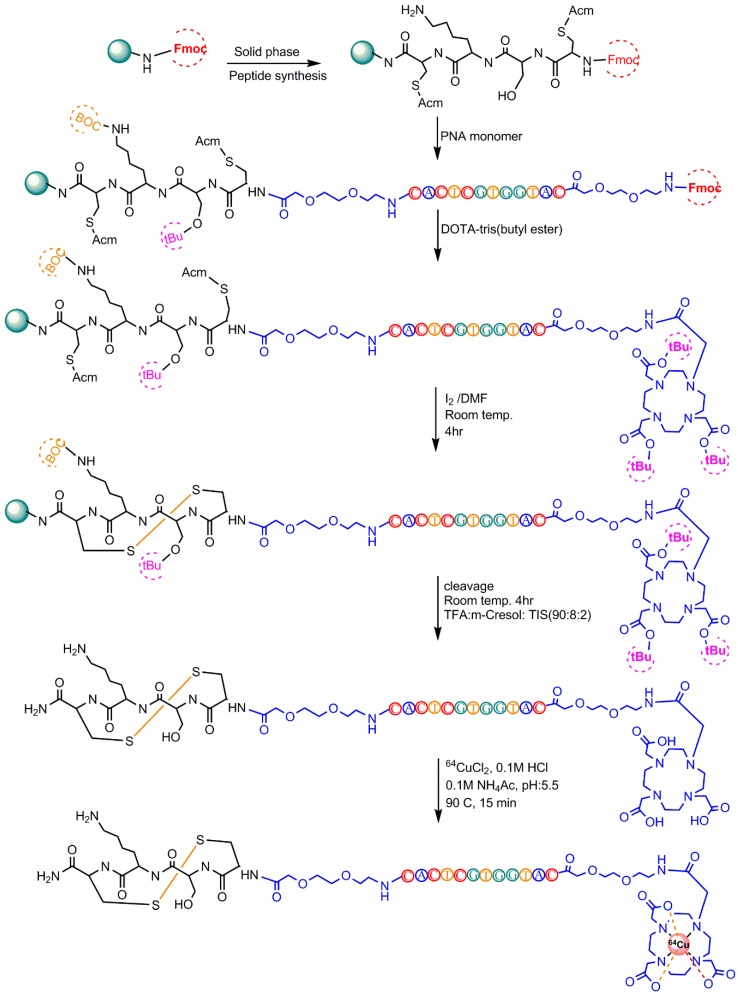
^64^Cu-radiolabeling steps of peptide nucleic acid.

Rockey et al. used these different chelators to radiolabel a 5′-amine-modified (12 carbon linker) RNA aptamer that binds PSMA (prostate specific membrane antigen) on prostate cancer cells. Three conjugation strategies were reported: DOTA via the NHS ester, NOTA and PCTA via the 2-S-(4-Isothiocyanatobenzyl (p–SCN–Bn) linker group, and 1,8-Diamino-3,6,10,13,16,19-hexaazabicyclo[6,6,6]-eicosane(diAmSar)via a disuccinimidyl suberate (DSS) linker [78]. Paudyal et al. designed and synthesized a radionuclide–chelator–peptide nucleic acid for detecting HER_2_ (Human Epidermal Growth Factor Receptor 2) mRNA in malignant breast cancer by PET imaging (Figure 16) [79]. Junling Li et al. reported the labeling of AS1411 (a 26-base guanine-rich oligonucleotide aptamer) with ^64^Cu for micro PET/CT study by conjugating four different chelators [80].

### 5.3. Zirconium-89 Labeling

#### 5.3.1. Desferrioxamine B Chelating Agent

**Figure 17:**
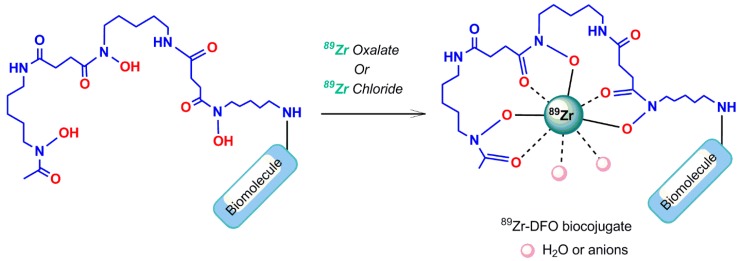
Conjugation of ^89^Zr to a Desferrioxamine B (DFO) bioconjugate.

Zirconium-89 (^89^Zr) is a positron emitter with a favorable half-life of 78.1 h, compared to other β-emitters, which allows long-term PET imaging. Because of its adequate half-life and positron emission, ^89^Zris used to radiolabel biomolecules for research in human and animal PET imaging, but it has not yet been approved for clinical applications [4]. Although direct labeling methods without using a chelator have been illustrated for ^89^Zr-introduction into serum proteins and liposomes, using an appropriate chelator system is a reasonable labeling approach for ^89^Zr [81,82]. ^89^Zr has not shown stable binding to DTPA [83], although it represents a higher stability than the comparable EDTA complex [84]. ^89^Zr complexation with Desferrioxamine B (a chelating agent exhibiting superior attributes compared to DTPA [83]) is still often used as a chelator for ^89^Zr-radiolabeling and is linked to the biomolecule before performing the radiolabeling reaction. The three hydroxamates and two additional anions or water molecules stabilize the Zr^4+^ ion, through the generation of an octadentate structural complex in the desferrioxamine B complex (Figure 17) [85].

#### 5.3.2. Hyperbranched Polymers (HBPs)

**Figure 18:**
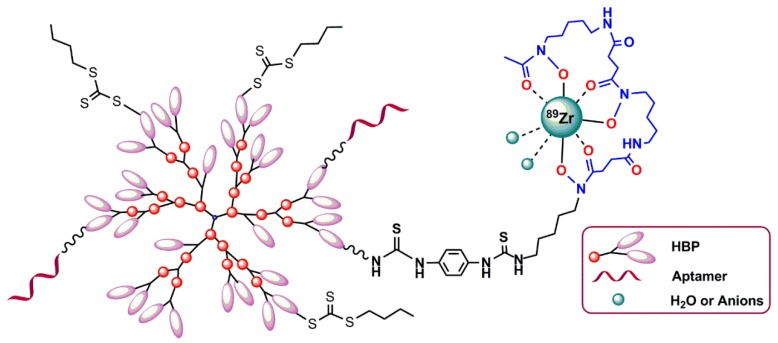
Oligonucleotide aptamer–hyperbranched polymer (HBP) conjugates as a targeted molecular imaging probe.

Hyperbranched polymers (HBPs) are the targeted molecular imaging probes, which incorporate both targeting and molecular imaging modalities within one structure [86,87,88]. Because of their several unique properties, such as tunable solution behavior, critical phase behavior, and presence of large numbers of functional end groups, HBPs are a highly interesting nanocarrier for drug delivery and molecular imaging. The chemical synthesis of different types of branched polymers is reported in the literature [89]. In 2016, Fletcher et al. reported the attachment of an oligonucleotide aptamer with HBPs targeting VEGF (the vascular endothelial growth factors) (VEGF), which are upregulated in triple-negative breast cancer. The attachment of an aptamer to polymer chain was performed by copper-free azide–alkyne 1,3-dipolar cycloaddition. The aptamer–HBP complex was then functionalized with a ^89^Zr chelator (desferoxamine) to enable PET imaging with ^89^Zr (Figure 18) [90].

### 5.4. Gallium-68 (^68^Ga) Radiolabeling

**Figure 19:**
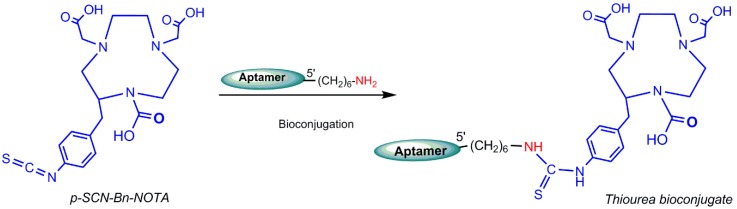
Amine–isothiocyanate bioconjugation reaction of isothiocyanate (SCN)–Bn–1,4,7-triazacyclononane–*N*,*N*′,*N*′′–triacetic acid (NOTA) to a hexylamine-functionalized oligonucleotide.

**Figure 20:**
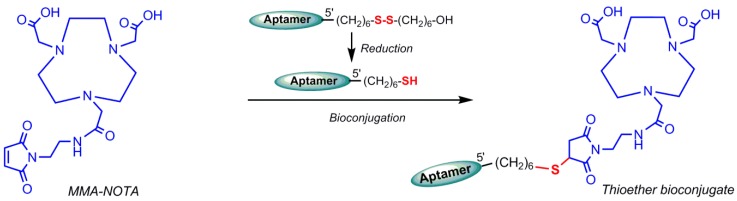
Thiol–maleimide bioconjugation reaction of maleimidoethylmonoamide(MMA)–NOTA to a hexylthiol-functionalized oligonucleotide.

Germanium-68 (^68^Ge) (t_1/2_ = 270.8 days) decays to ^68^Ga and ^68^Ga (t_1/2_ = 68 min) disintegrates by positron emission, which is used in PET imaging. Owing to the approximate half-life duration difference, the Germanium-68/Gallium-68 generator is manufactured in cyclotron and commercially available in nuclear medicine centers. The ^68^Ge/^68^Ga generator is approved in Europe for clinical use, particularly for the preparation of ^68^Ga–DOTATOC (TOC = a peptide like Tyr3–octreotide) and ^68^Ga–DOTATATE (TATE = Tyr3–octreotate) for neuroendocrine tumor detections [4]. In addition, ^68^Ga is considered as an attractive theranostic radionuclide because of its ability to be substituted by therapeutic radionuclides, such as ^90^Y or ^177^Lu [91]. Generator accessibility, simple and rapid labeling, and kit formulations are the advantages of ^68^Ga over ^18^F, the leading radionuclide for PET imaging [92]. ^68^Ga radiolabeling can be performed by complexation of ^68^Ga^3+^ cation by a potent chelator. Owing to commercial availability and well-characterized coordination chemistry, DOTA is the gold standard for ^68^Ga radiolabeling. Gallium (III) complex of NOTA ((1,4,7-triazanonane–1,4,7-triyl)triacetic acid) is thermodynamically more stable than DOTA and radiolabeling of NOTA can be achieved at room temperature under mild conditions compatible with most biomolecules [93]. Gijs M et al. in 2016 reported the ^68^Ga-radiolebelled HER_2_ aptamers [88]. A HER_2_-non-specific DNA oligonucleotide (5′-CCC TTT TAC ACA ACC ATC GAC ATA ACT AAA ACC ACC ACT G-3′), which was functionalized at its 5′-end with hexyldisulfide or hexylamine, was conjugated with p–SCN–Bn–NOTA (Figure 19 and Figure 20) at room temperature in the dark. Aptamers with disulfide functional groups were conjugated at inert atmosphere (argon) to avoid reoxidation in air. The main advantage of these bioconjugation approaches is that there is only one binding site for the chelator [94].

**Figure 21:**
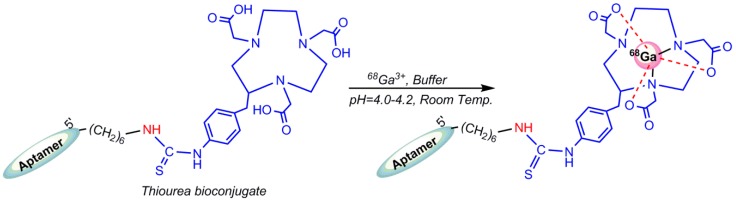
^68^Ga-radiolabeling of Thiourea–NOTA–aptamer.

^68^Ga-radiolabeling of bioconjugate (solubilized in sodium acetate or a (4-(2-hydroxyethyl)-1-piperazineethanesulfonic acid) HEPES buffer) was performed by addition of pre-concentrated ^68^Ga^3+^ eluate and incubation at room temperature. The author has pointed the low accessibility of primary amine group by three-dimensional structure of oligonucleotide as the reason for the low bioconjugation yields of the mentioned conjugation, as shown in Figure 19. Unlike amine–isothiocyanate-based reactions, the thiol–maleimide-based reaction is not reversible, so that the reducing agents are not be able to cleave the bond. The radiolabeling process of oligonucleotides should be conducted in nearly neutral pH circumstances to prevent acid-catalyzed depurination or hydrolysis incidents. Meanwhile, ^68^Ga^3+^ is stable only under acidic (pH < 3) conditions. ^68^Ga^3+^ creates insoluble Ga(OH)_3_ (gallium hydroxide) complexes at pH between 3 and 7, owing to strong affinity for hydroxide anions [95]. Radiolabeling of conjugated HER_2_ aptamer was performed using a lyophilized aptamer in a HEPES buffer and incubated for at least 5 min at room temperature (Figure 21) [96]. In 2015, Kang et al. reported ^68^Ga labeling of uMUC-1 (underglycosylated mucin-1, an early marker of tumor development) targeting an aptamer using a p–SCN–bn–NOTA chelating agent [97].

## 6. Conclusions and Future Prospects

Nucleic acid aptamers show high binding affinity to molecular targets with high specificity, which makes them effective biomolecules for generating excellent molecular imaging agents. They require functionalization to generate target-specific molecular imaging probes [98]. One of the most popular probe design approaches is the incorporation of functional groups at the 5′ or 3′ ends of aptamers, such as thiols or amines, via phosphoramidite reaction during aptamer synthesis [99]. Despite their many advantages as targeting agents, aptamers possess susceptibility to endogenous nuclease degradation in vivo, and common strategies to overcome this limitation are to enhance nuclease stability by the incorporation of non-natural nucleic acids, such as locked nucleic acids [100,101,102].

Molecular imaging by aptamers in nuclear tomographic imaging usually requires the covalent attachment of a chelator to the 5′ terminal amine of an oligonucleotide aptamer, and then the chelators conjugate radionuclides and it is feasible to proceed with nuclear imaging of the molecular target [98]. In this review, we highlight the most frequently utilized chelators and radionuclides in recent years for generating molecular imaging probes of aptamers, such as SPECT and PET imaging agents used in recent years have been highlighted. In general, aptamer functionalization may compromise its target affinity. However, in line with the reported literature discussed in this review, it is worth mentioning that radiolabeling of aptamers did not affect the actual aptamer target binding affinity. The literature indicates an enhancement of aptamer-targeted SPECT and PET probes and the recent promising results obtained in aptamer-targeted nuclear imaging probes will soon proceed to clinical applications, particularly in the oncology field.

## Figures and Tables

**Table 1:** Characteristics of common nuclear medicine radionuclides [4,20].

Radioisotopes	Atomic Number	Physical Half-Life	Decay Mode (%)
^11^C	6	20.4 min	β+(100)
^13^N	7	9.96 min	β+(100)
^15^O	8	2.03 min	β+(100)
^18^F	9	109.8 min	β+(97)
^62^Cu	29	9.76 min	β+(97), EC(3)
^64^Cu	29	12.8 h	β+ Or β−, EC
^67^Ga	31	3.3 days	EC(100)
^68^Ga	31	68 min	β+(89), EC(11)
^82^Rb	37	75 s	β+(95), EC(5)
^89^Zr	40	78.1 h	β+(23), EC(77)
^94m^Tc	43	52 min	β+(72), EC(28)
^99m^Tc	43	6.0 h	IT(100)
^111^In	49	2.8 days	EC(100)
^123^I	53	13.2 h	EC(100)

β+(Positron), EC (Electron Capture), IT (Isomeric Transition).
